# Risk factors for unsuccessful tuberculosis treatment outcome (failure, default and death) in public health institutions, Eastern Ethiopia

**DOI:** 10.11604/pamj.2015.20.247.3345

**Published:** 2015-03-16

**Authors:** Tariku Dingeta Amante, Tekabe Abdosh Ahemed

**Affiliations:** 1 Department of Public Health, College of Health and Medical Sciences, Haramaya University Harar, Ethiopia; 2 School of Medicine, College of Health and Medical Sciences, Haramaya University, Harar, Ethiopia

**Keywords:** Unsuccessful TB treatment outcomes, sputum smear negative, death

## Abstract

**Introduction:**

Unsuccessful TB treatment outcome is a serious public health concern. It is compelling to identify, and deal with factors determining unsuccessful treatment outcome. Therefore, study was aimed to determine pattern of unsuccessful TB treatment outcome and associated factors in eastern Ethiopia.

**Methods:**

A case control study was used. Cases were records of TB patients registered as defaulter, dead and/or treatment failure where as controls were those cured or treatment complete. Multivariate logistic regression models were used to derive adjusted odds ratios (OR) at 95% CI to examine the relationship between the unsuccessful TB treatment outcome and patients’ characteristics.

**Results:**

A total of 990 sample size (330 cases and 660 controls) were included. Among cases (n = 330), majority 212(64.2%) were because of death, 100(30.3%) defaulters and 18(5.5%) were treatment failure. Lack of contact person(OR = 1.37; 95% CI 1.14-2.9, P, .024), sputum smear negative treatment category at initiation of treatment (OR = 1.8; 95% CI 1.3-5.5,P, .028), smear positive sputum test result at 2^nd^ month after initiation treatment (OR = 14; 95% CI 5.5-36, P,0.001) and HIV positive status (OR = 2.5; 95% CI 1.34-5.7, P, 0.01) were independently associated with increased risk of unsuccessful TB treatment outcome.

**Conclusion:**

Death was the major cause of unsuccessful TB treatment outcome. TB patients do not have contact person, sputum smear negative treatment category at initiation of treatment, smear positive on 2^nd^ month after treatment initiation and HIV positive were factors significantly associated unsuccessful treatment outcome. TB patients with sputum smear negative treatment category, HIV positive and smear positive on 2^nd^ nd month of treatment initiation need strict follow up throughout DOTs period.

## Introduction

Tuberculosis (TB) is a major cause of illness and death worldwide. It is one of the leading causes of morbidity and death in sub- Saharan African countries. Ethiopia ranks 3^rd^ among sub-Saharan African countries. The burden is exacerbated by the spread of HIV infection [1, 2]. In Ethiopia free TB diagnosis and treatment is undertaking at 1,448 state-owned health service institutions and more than 230 private health facilities. While treatment is integrated into general health services and DOTS geographical coverage is 95%, TB remains a major health problem in Ethiopia. Cure rate of 67% remains well below the 85% rate of WHO recommendation [3]. A retrospective study from north Ethiopia also depicts the treatment success rate of tuberculosis patients was unsatisfactory [4]. An earlier study on the impact of DOTS in the Southern Ethiopia reported one in five TB patients still continued to result with unsuccessful treatment outcome. Default is one of the unsuccessful forms of TB treatment outcome and a serious problem in the TB program of Ethiopia. According to the retrospective study in rural hospital in South Ethiopia defaulting from treatment rate was 11.4%. Another study from Northern part of the Country also reviled among unsuccessful treatment outcome, 18.3% were defaulted followed by death and treatment failure account 10.1% and 0.2% respectively [4–6].

In effort to reach the global target of 85% treatment success, it is compelling to identify, describe, and deal with factors determining poor treatment outcome. Several reasons and risk factors for unsuccessful TB treatment outcomes have been reported from different countries [4, 5, 7, 8]. However, up to the researches awareness it is not clear which factors are major contributors to the unsuccessful TB treatment outcome of TB patients in the eastern part of Ethiopia. Failure to treatment completion or cure is believed to be the main reason for difficulties in controlling a disease that is far from new. For these reasons, determination of the pattern of unsuccessful treatment outcome and factors that predicts the unsuccessful treatment outcomes helps to design the possible future of TB treatment and control in the community. Therefore, this study was aimed to describe pattern of unsuccessful TB treatment outcome and associated factors among health institutions providing DOTS in eastern Ethiopia.

## Methods

### Settings and study design

Intuitional based case control study design was conducted using the patients’ record in 6 TB clinics providing DOTS in East Hararge of Oromia Region, Dire Dawa Administration and Harari regional State, Eastern Ethiopian. Cases were those registered as defaulter, death or treatment failure on the TB registration log book. For each case, 2 controls enrolled in the same week or one week later or earlier and declared as cured or treatment complete were selected. Data was collected from September 1^st^ to October 30, 2012.

### Sample Size and Sampling Procedures

Sample size was calculated based on the assumption previous treatment defaulter as one of the important risk factor for unsuccessful treatment outcome, 5% level of significance and 80% power. In an earlier study, we found that the proportion of success full treatment outcome among started treatment after defaulter in southern Ethiopia is 46% where as 55% of patient return after default result with unsuccessful treatment outcome [7]. Based on the above assumption the final sample size was 330 cases and 660 controls with 95% certainty that return after default is a statistically significant risk factor for not to complete treatment successfully. Records of TB patients from September 2007 to August 30, 2012 were reviewed. The total sample size was proportionally allocated for each institutions based on the total patient started TB treatment in the last five years before the data collection time. Additionally, the total sample size allocated for each institution was also allocated for each Ethiopian Fiscal year based on the total number of TB patient started treatment during each year. All the list of defaulter, dead or treatment failure patients was reviewed until the sample size was fulfilled. To improve the comparison of cases and controls for one case two controls that started the treatment in the same week in the same institution was included. If more than two controls started in the same week; the one with the closest recoded with the case was selected.

### Data Collection

Data was collected using a check list prepared from TB registration logbook form patient records, which was developed by the Ministry of Health. They were filled out by health workers working in the TB clinics. To ensure the quality of data, pre-test of data collection tools was done in one hospital and one health centre not included in the study. Training was given for data collectors and data collection process was supervised by investigators. Every check list was checked for its completeness during data collection.

### Data Analysis and Processing

The collected quantitative data was coded by investigators and entered to Epi data and transferred to SPSS Version 17.0. for analysis. Descriptive statistics was carried out to explore the background characteristics. A stepwise logistic regression model was used to examine the relationship between the outcome variables and selected determinant factors. Variables recorded on the TB registration logbook were included in the logistic regression to determine predictors of unsuccessful TB treatment outcome. Odds ratio (OR) with 95% CI was calculated as a measurement of association. P-values equal to or below 0.05 were considered statistically significant.

### Ethical Considerations

The study protocol was approved by Institutional Research Ethics Review Committees of the College of Health Sciences of Haramaya University. In order to protect the confidentiality of the information, names or IDs was not included in data extraction check list.

## Results

### Socio-demographic Characteristics

Records of 330(33.8%) cases and 646(66.2%) controls with a total of 976 patient records were reviewed. Majority were female (63.6% case vs 57.9% controls). Majority of the patient included(59.3% cases Vs 61% controls) were found within age group of 20-34 years with mean age of 32.23(34.9 years among cases vs 30.9 years among controls). Moreover, more than half (57.3% cases vs 71.5% controls) had contact person. From patient included in the analysis there were more likely to have HIV test (68.4% of cases and 24.2% f controls). Among the total of study participants get the HIV test (n = 641) 35.9% of cases and 24.2% of controls (P< 0.002) were HIV positive (Table 1).

**Table 1:** Characteristics of patients started DOTS in selected health institutions in eastern Ethiopia from September 2007 to August 30, 2012

Variables	Frequency (%)	
	Case(Unsuccessful)	Control(Successful)
**Sex**		
Male	210(63.6)	374(57.9)
Female	120(34.4)	272(42.1)
**Age in Year**		
9 and below	18(5)	44(6.8)
10-19	26(7.9)	82(12.7)
20-29	94(28.6)	199(30.9)
30-34	101(30.7)	194(30.1)
45 and above	90(27.4)	125(19.4)
**Have contact person**		
Yes	189(57.3)	462(71.5)
No	141(42.7)	184(28.5)
HIV test offered		
Yes	206(68.4)	435(24.2)
No	95(31.6)	166(75.8)
**If HIV test result**		
Sero positive	74(35.9)	105(24.2)
Sero negative	132(64.1)	328(75.8)
**Treatment category at the time of treatment initiation**		
New	282(85.5)	569(88.1)
Relapse	24(7.3)	35(5.4)
Treatment Failure	5(1.5)	2(0.3)
Defaulters	6(1.8)	21(3.3)
Transfer in	13(3.9)	19(2.9)
**Diagnosis Category**		
Pulmonary Positive(P/Pos)	112(33.6)	335(49.8)
Pulmonary negative (P/Neg)	150(47.3)	205(32)
Extra pulmonary (EP)	68(19.1)	106(8.1)
**Sputum AFB result at 2^nd^month after initiation of treatment**		
Positive	17(35.4)	13(4%)
Negative	31(64.6)	314(96)

### TB Treatment related Characteristics

Among the cases (n = 330), majority 212(64.2%) were because of death and followed by those who were defaulters 100(30.3%) and the rest 18(5.5%) were because of treatment failure. Among the total patients, most of them 802(82.2%) were pulmonary Tuberculosis (PTB) (80.9% of cases Vs 81.8% of controls). From the total of pulmonary Tuberculosis (PTB) patients, 447(55.7%) were smear negative PTB (47.3% of cases and 32% of controls, P < 0.001). Of the smear positive TB patients, 13(4.2%) were smear positive again at the 2nd month after starting treatment with significant difference between cases and controls (35.4% of cases Vs 4% of controls, P < 0.001). About 851(87.2%) of the study participants were new treatment category (85.5% of cases and 88.1% of controls). About 93(9.5%) (10.6% of cases and 9% of controls) had history of previous unsuccessful full treatment outcome. Among the 9.5% with previous history of unsuccessful treatment outcome, 6% were because of relapse (7.3% of cases and 5.4% of controls), 2.8% were secondary to defaulter (1.8% of cases vs 3.3% of controls) and the rest 0.7% were secondary to treatment failure (with 1.5% of cases and 0.3% of controls) (Table 1).

### Factors Associated with unsuccessful outcome of TB treatment

To evaluate any factors associated with unsuccessful TB treatment outcome gender, age, presence of contact person, diagnosis category (smear positive pulmonary TB, smear negative Pulmonary TB, Extra Pulmonary TB), treatment category (new vs previously unsuccessful), HIV sero- status and sputum result at 2^nd^ month after initiation of treatment were entered into multiple logistic regression analysis. Lack of contact person to be contacted at a time of treatment interruption is one of the predictor of higher unsuccessful treatment outcome (AOR 1.37; 95% CI 1.14-2.91, P = 0.024). Sputum smear negative diagnosis is also another risk factors that predict the unsuccessful treatment outcome (OR 1.83; 95% CI 1.3-5.51, =0.028). HIV positive status (OR 2.3; 95% CI 1.34-5.73, P= 0.002) and positive sputum test result at 2^nd^ month after initiation of treatment (OR 14.2; 95% CI 5.52-36.46, P< 0.001) are also independently associated high odds of unsuccessful outcome (Table 2).

**Table 2:** Factors associated with unsuccessful TB treatment outcome among selected health institution in eastern Ethiopia, 2013

Variables	Unsuccessful Treatment outcome		
	COR	AOR	P-Value
**Sex**			
Male	1.3(0.97-1.7)	1.05(0.53-2.20)	0.88
Female	1.00	Reference	
Age			
15 and less	0.53(0.32-0.89)	0.8(0.14-3.90)	0.7
16-44	0.66(0.48-0.90)	1.3(0.23-7.60)	0.18
45 and above	1.00	Reference	
**Have contact person**			
No	1.87(1.42-2.47)	1.37(1.14-2.91)	0.024
Yes	1.00	Reference	
**Sputum smear result at the beginning of treatment**			
Smear Negative	2.2(1.6-2.95)	1.83(1.3-5.51)	0.028
Extra pulmonary(EPT)	1.4(0.96-2.02)	1.54(0.7-4.39)	0.155
Smear positive	1.00	Reference	
**Treatment category**			
Previously unsuccessful	1.3(0.9-1.85)	0.94(0.34-2.60)	0.09
New	1.00	Reference	
**HIV Sero-status**			
Positive	1.81(1.32-2.52)	2.53(1.34-5.73)	0.01
Negative	1.00	Reference	
**Sputum AFB result at 2^nd^ month after initiation of treatment**			
Positive	13.25(5.90-29.80)	14.23(5.52-36.46)	0.001
Negative	1.00	Reference	

## Discussion

This study describes the characteristics of patients with TB and their Treatment outcome over a past 5-years period in a selected health institutions in eastern Ethiopia. Our finding shows among the participant with unsuccessful treatment outcome majority 212(64.2%) were because of death. Out of the total reviewed patients′ record more proportion of patient with unsuccessful treatment outcome were pulmonary negative compared to those successfully completed the treatment (47.3% of cases and 32% of controls, P < 0.001). This study also demonstrated positive HIV sero-status, positive sputum test result at 2nd month after initiation of treatment, negative sputum smear pulmonary TB diagnosis category at the begging of treatment and lack of contact person were the factors significantly associated with unsuccessful treatment outcome after adjusted for other variables. Among the total of patient with unsuccessful treatment outcome (n = 330) 64.2% were because of death. A study from Taipei, Taiwan among adult also showed death was the main cause of unsuccessful treatment outcome of Tuberculosis patients [8]. Contrary similar study conducted in North Ethiopia showed defaulter was more cause of unsuccessful treatment outcome than death [4]. The difference might be related with starting of using strategies such as health extension workers to increase public awareness on the importance of effective completion of TB treatment; use of phone of contact person to ascertain reason for lost follow up are being undertaken so that might contribute to the decreasing of defaulter from TB treatment. The main concern about TB/HIV co-infection is that, different study result from different part of the world showed the TB treatment outcome probability are significantly different between HIV+ and HIV negative TB patients. The prevalence of HIV co-infection was 35.9% among patients with unsuccessful treatment outcome which is 24.2% among successfully complete treatment. This showed the risk of unsuccessful TB treatment outcome is significantly high among patients with HIV positive compared to HIV negative sero status (OR= 2.33(1.34-5.73, P < 0.01). It is comparable with many studies that have found that HIV co-infections are associated with poorer outcomes compared to HIV-negative patients [9–13]. One can understand that HIV is continued to be double challenge both increasing the risk of developing and poor outcome to treatment of TB.

Sputum test positive for AFB result at 2nd month after initiation of treatment was significantly high among unsuccessful treatment outcome compared to those successful treatment outcome (OR= 14.23(5.52-36.46). This result is in line with a study from Yunnan, China which showed positive 2-month smear test result is one of the risk factors for unsuccessful treatment outcome [14, 15]. Our study showed sputum smear negative pulmonary TB at the begging of treatment is a factor that predict unsuccessful TB treatment outcome (OR 1.83, 95% CI 1.3-5.51). The finding is in line with previous studies from Ethiopia and other countries [4, 8]. This might because of probability of miss diagnosis of the patients which resulted with poor treatment response. This study also depicts lack of registered contact person was also the risk factor associated with unsuccessful treatment outcome (OR = 1.37(1.14-2.91, p < 0.024). There are some limitations to this study. First, the retrospective nature of the study is a methodological limitation. Second, we used only routine programme data; so that the Ethiopian health institution TB log book might has no enough check list to identify full history of TB patients′ characteristics, thus it is possible that there might be another associated factors left to examined. The analysis can only provide evidence of statistical association between those items and unsuccessful treatment outcome and cannot show cause-effect relationships. Despite these limitations, the study findings are useful to inform policy and programmers that aim to improve management of TB patients in Ethiopia and other comparable settings.

## Conclusion

In summary, our study has provided us with useful insights on factors influencing unsuccessful treatment outcome. Death was the major unsuccessful treatment outcome of tuberculosis patients in the study area. AFB smear positive on 2^nd^month after initiation of treatment was significantly associated high unsuccessful treatment outcome. Our results also confirmed previous findings TB/HIV co-infection is factor associated with increased risk of unsuccessful treatment outcome. Lack of contact person and negative sputum smear pulmonary TB diagnosis at the begging of treatment are also the factors significantly associated with high unsuccessful treatment outcome. Based on the results, we recommend HIV positive TB patients and sputum smear positive at 2^nd^ month after initiation TB treatment need strict follow up throughout the DOTs period. Moreover, even though DOTS is only for intensive cases those became smear positive on 2^nd^ month after initiation TB treatment should be considered for follow up on daily basis by health professionals. Prospective study is needed in the study area to identify main cause of unsuccessful outcome.

## References

[1] WHO (2008). Global Tuberculosis Control - Surveillance, Planning, Financing.

[2] WHO Ethiopia Tuberculosis profile. http://www.who.int/tb/data.

[3] USAID (2010). Global Health Initiative, Ethiopia Strategy.

[4] Tessema B, Muche A, Bekele A, Reissig D, Emmrich F, Sack U (2009). Treatment outcome of tuberculosis patients at Gondar University Teaching Hospital, Northwest Ethiopia: a five - year retrospective study. BMC Public Health.

[5] Manuel JR, Reyes F, Tesfamariam A (2010). Childhood and adult tuberculosis in a rural hospital in Southeast Ethiopia: a ten-year retrospective study. BMC Public Health..

[6] Biru E, Lindtjørn B (2007). Determinants of Treatment Adherence Among Smear-Positive Pulmonary Tuberculosis Patients in Southern Ethiopia. PLoS Medicine..

[7] Shargie BE, Lindtjørn B (2005). DOTS improve treatment outcomes and service coverage for tuberculosis in South Ethiopia: a retrospective trend analysis. BMC Public Health..

[8] Yen Y-F, Yen M-Y, Shih HC, Deng ChY (2012). Risk factors for unfavorable outcome of pulmonary tuberculosis in adults in Taipei, Taiwan. Transactions of the Royal Society of Tropical Medicine and Hygiene..

[9] Shaweno D, Workua A (2012). Tuberculosis treatment survival of HIV positive TB patients on directly observed treatment short-course in Southern Ethiopia: A retrospective cohort study. BMC Research Notes.

[10] Tweya H, Feldacker C, Phiri S, Ben-Smith A, Fenner L, Jahn A, Kalulu M, Weigel R, Kamba C, Banda R, Egger M, Keiser O (2013). Comparison of Treatment Outcomes of New Smear- Positive Pulmonary Tuberculosis Patients by HIV and Antiretroviral Status in a TB/HIV Clinic, Malawi. Plos One..

[11] Lorent N, Sebatunzi O, Mukeshimana G, Ende JV, Clerinx J (2011). Incidence and Risk Factors of Serious Adverse Events during Antituberculous Treatment in Rwanda: A Prospective Cohort Study. PLoS ONE..

[12] Zhuy T, Shah NS, Anh MH, Nghia DT, Thom D, Linh L, Ngoc Sy D, Duong BD, Minh Chau LT, Phuong Mai PT, Wells CD, Laserson KF, Varma JK (2007). HIV-Associated TB in An Giang Province, Vietnam, 2001-2004: Epidemiology and TB Treatment Outcomes. PLoS ONE..

[13] Pefura Yone EW, Kuaban Ch, Kengne AP (2012). HIV testing, HIV status and outcomes of treatment for tuberculosis in a major diagnosis and treatment centre in Yaounde, Cameroon: a retrospective cohort study. BMC Infectious Diseases.

[14] Jianzhao H, Hof S, Lin X, Yubang Q, Jinglong H, Werf MJ (2011). Risk factors for non-cure among new sputum smear positive tuberculosis patients treated in tuberculosis dispensaries in Yunnan, China. BMC Health Services Research..

[15] Pardeshi GS, Deshmukh D (2007). A comparison of treatment outcome in re-treatment versus new smear positive cases of tuberculosis under RNTCP. Indian J Public Health..

